# Respiratory Muscle Strength in Hypertensive Adults: Age- and Sex-Specific Reference Values for a Brazilian Population

**DOI:** 10.3390/medsci13040323

**Published:** 2025-12-17

**Authors:** Simone de Faria Rossetto, Juscelio Pereira da Silva, Afonso Santos de Lima, Anderson Geremias Macedo, Dalton Muller Pessôa Filho, Albená Nunes Silva, Thiago Roberto Lima Romero, Giovane Galdino

**Affiliations:** 1 Cardiovascular Rehabilitation Sector, Institute of Motor Sciences, Federal University of Alfenas (UNIFAL), Alfenas 37131-001, MG, Brazil; simonefariarossetto@hotmail.com (S.d.F.R.); afonso.sd.lima@gmail.com (A.S.d.L.); 2 Pos-Graduation Program in Rehabilitation Sciences, Institute of Motricity Sciences, Federal University of Alfenas (UNIFAL), Santa Clara Campus, Alfenas 37133-840, MG, Brazil; anderson.macedo@unifal-mg.edu.br; 3 Department of Physiotherapy, Federal University of Minas Gerais, Belo Horizonte 31270-901, MG, Brazil; juscelio.silva@unifal-mg.edu.br; 4 School of Sciences, São Paulo State University (UNESP), Bauru 17033-360, SP, Brazil; dalton.pessoa-filho@unesp.br; 5 Graduate Program in Human Development and Technology, São Paulo State University (UNESP), Rio Claro 13506-900, SP, Brazil; 6 Inflammation and Exercise Immunology Laboratory, Federal University of Ouro Preto, Ouro Preto 35400-000, MG, Brazil; albenanunes@hotmail.com; 7 Laboratory of Pain and Analgesia, Pharmacology Department, Federal University of Minas Gerais, Belo Horizonte 31270-901, MG, Brazil; thiromero@gmail.com

**Keywords:** respiratory muscle strength, maximal inspiratory pressure, maximal expiratory pressure, hypertension

## Abstract

Background/Objectives: Hypertension is a major modifiable risk factor for cardiovascular disease and may negatively affect muscle strength through vascular and metabolic mechanisms. Nevertheless, reference values for respiratory muscle strength (RMS) in hypertensive adults remain unavailable. This study aimed to establish sex- and age-specific reference values for maximal inspiratory pressure (MIP) and maximal expiratory pressure (MEP) in Brazilian adults with hypertension and to investigate clinical factors associated with these measures. Methods: This cross-sectional study included 234 hypertensive adults (109 women and 125 men; 30–80 years) enrolled in a cardiovascular rehabilitation program. Anthropometric, hemodynamic, and clinical data were collected. RMS was assessed using standardized procedures for MIP and MEP with an analog manovacuometer (−300 to +300 cmH_2_O). Participants were stratified by age and sex. Statistical analyses included the Mann–Whitney U and Kruskal–Wallis tests and multivariate linear regression (*p* < 0.05). Results: Men exhibited significantly higher MIP and MEP values than women across most age groups. RMS declined progressively with age, with a more marked reduction after 60 years. MIP was identified as the primary predictor of MEP in both sexes, with higher coefficients of determination in men. The reference values obtained were largely comparable to those reported for healthy individuals. Conclusions: This study provides clinically relevant reference values for RMS Brazilian individuals with hypertension, offering useful parameters for respiratory assessment and individualized rehabilitation strategies.

## 1. Introduction

Hypertension is widely recognized as the leading modifiable risk factor for cardiovascular disease (CVD) and all-cause mortality worldwide. Its considerable public health burden stems not only from its high prevalence but also from its well-established association with adverse clinical outcomes, including stroke, coronary artery disease, heart failure, and chronic kidney disease [1].

Beyond its relationship with multiple comorbidities, hypertension has also been associated with reduced muscle strength, as reported in recent studies [2,3,4]. Notably, Bai et al. [2] demonstrated that muscle quality—defined as muscle strength relative to muscle mass—is inversely associated with hypertension. Although muscle strength is a critical parameter to assess in individuals with hypertension, particularly given its relevance to conditions such as sarcopenia and increased mortality risk, there remains a marked paucity of research specifically addressing respiratory muscle strength (RMS) in this population.

RMS, defined as the maximal force generated by the respiratory muscles during inspiration and expiration, plays a crucial role in optimizing athletic performance and preserving respiratory health, particularly in individuals with pulmonary or neuromuscular disorders [5]. Evidence indicates that enhancing RMS can improve physical performance in both athletes and healthy individuals, as well as functional capacity in patients with respiratory or systemic diseases [6,7,8,9,10]. In hypertensive populations, recent findings show that inspiratory muscle training improves exercise capacity, physical function, peripheral muscle strength, and resting dyspnea [10]. Moreover, a recent meta-analysis demonstrated that respiratory muscle training effectively reduces systolic and diastolic blood pressure, as well as resting heart rate, in individuals with hypertension.

Although respiratory muscle training has been shown to be effective and beneficial in hypertensive individuals, baseline RMS values in this population remain poorly characterized. One of the primary methods used to assess RMS is manovacuometry, a simple, non-invasive, and easily applicable technique that measures inspiratory and expiratory muscle strength through maximal inspiratory pressure (MIP) and maximal expiratory pressure (MEP), respectively. Manovacuometry has been widely employed in both clinical and research settings [11,12,13]. Therefore, MIP and MEP values serve not only as indicators for determining the appropriate training load for respiratory muscle conditioning but also as objective measures of the RMS of the individual being evaluated.

Therefore, the present study aimed to establish reference values for MIP and MEP in hypertensive individuals across different age groups and sexes, and to investigate the influence of relevant clinical factors on these parameters.

## 2. Materials and Methods

### 2.1. Study Population

This cross-sectional observational study was conducted at the Cardiovascular Rehabilitation Unit of the Federal University of Alfenas (UNIFAL), Brazil, and is reported in accordance with the Strengthening the Reporting of Observational Studies in Epidemiology (STROBE) guidelines [14]. A total of 305 individuals diagnosed with hypertension were enrolled in the UNIFAL Cardiovascular Rehabilitation Program between 1 January 2013, and 31 August 2019. All procedures described in this manuscript were performed in accordance with the ethical principles of the Declaration of Helsinki. The study protocol was approved by the Human Research Ethics Committee of the Federal University of Alfenas (UNIFAL) (CAEE 09381018.6.0000.5142; approval number 3.262.183; approved on 12 April 2012).

Participants aged 30 to 80 years, of both sexes, and diagnosed with either primary or secondary systemic arterial hypertension were eligible for inclusion. Exclusion criteria included the presence of respiratory or pulmonary diseases; musculoskeletal or neurological disorders (including sequelae) that limited aerobic exercise; acute inflammatory or infectious conditions; chronic pain; a history of neoplasia within the previous five years; use of immunosuppressive agents; lower-limb amputations; recent lower-limb surgery or fractures (within the past six months); unstable angina; recent thrombophlebitis or thromboembolism; third-degree atrioventricular block; myocarditis; pericarditis; uncontrolled cardiac arrhythmias; decompensated heart failure; and persistent uncontrolled hypertension despite pharmacological treatment.

Participants included in the study were individuals evaluated prior to initiating a cardiovascular rehabilitation program. This setting was selected because it provides standardized and routinely performed clinical and physiological assessments, including measurements of respiratory muscle strength, which are not systematically collected in the general hypertensive population. Hypertension was the most prevalent condition among those admitted to the program, allowing the formation of a sufficiently large and homogeneous sample for the development of reference values. As described previously, individuals with respiratory, neuromuscular, or other clinical conditions that could directly influence respiratory muscle performance were excluded to minimize potential confounding factors and ensure that the findings reflected characteristics specific to hypertensive patients.

### 2.2. Data Collection

All data for this study were obtained during the initial assessment performed at each patient’s admission to the rehabilitation unit by a specialized physiotherapist. Patients who had previously undergone physical rehabilitation, engaged in regular physical activity, or received similar interventions were not included. The assessment consisted of collecting personal and clinical information, including medical history (past and current conditions), lifestyle habits, comorbidities, and ongoing pharmacological treatments. Subsequently, a series of physical evaluations and tests was conducted, as detailed below.

### 2.3. Procedures

The primary outcome of this study was to establish reference values for RMS in individuals with hypertension. Secondary outcomes included examining the effects of age and sex on these values. Accordingly, the following procedures were implemented to assess these outcomes.

Height and body mass were measured to calculate body mass index (BMI). Height was assessed using a portable stadiometer (Seca 217, Hamburg, Germany) with participants standing barefoot, upright, with arms relaxed at their sides, and with heels, back, and head aligned against the vertical support of the device. Body weight was measured in kilograms using a platform scale with a 200-kg capacity and 100-g precision, positioned on a flat and calibrated surface before each assessment. Nutritional status was classified according to World Health Organization criteria [15] based on BMI, calculated as weight (kg)/height (m^2^).

Additionally, waist circumference (WC), systolic blood pressure (SBP), diastolic blood pressure (DBP), and resting heart rate (HR) were assessed. WC was measured using a flexible, non-elastic tape placed at the midpoint between the lower margin of the last palpable rib and the top of the iliac crest, with participants standing upright, abdomen relaxed, arms at their sides, and feet together. SBP and DBP were measured after participants had been seated at rest for at least 10 min. Mid–upper arm circumference was measured to determine the appropriate cuff size. Blood pressure was then assessed on the left arm, positioned at heart level, using a calibrated aneroid sphygmomanometer with the correctly sized cuff. Three consecutive measurements were obtained, and the mean value was used for analysis. Standardized protocols for cuff selection, participant positioning, and measurement procedures were strictly followed [16]. Resting HR was measured by palpating the radial artery for 60 s using a wristwatch, after a minimum of five minutes of seated rest.

Following the anthropometric and clinical evaluations, respiratory muscle strength (RMS) was assessed to determine maximal inspiratory and expiratory pressures. RMS was measured using a calibrated analog manovacuometer (M120, Globalmed, Porto Alegre, Brazil) with an operating range of −300 to +300 cmH_2_O and scale increments of 4 cmH_2_O. Prior to testing, participants received standardized verbal instructions and a brief demonstration to ensure proper execution of the maneuvers.

Accordingly, maximal inspiratory pressure (MIP) was measured by instructing participants to perform a maximal inspiratory effort starting from residual volume, whereas maximal expiratory pressure (MEP) was assessed following a maximal expiratory effort from total lung capacity. Each maneuver was repeated three times, and the highest value was recorded for analysis. All pressure efforts were sustained for approximately one second under verbal guidance, with a one-minute rest interval between attempts [17]. The measured MIP and MEP values were subsequently compared with the predicted reference values for the Brazilian population proposed by Neder et al. [17].

To ensure consistency across all procedures, all measurements were performed between 7:30 and 10:00 a.m. Participants were instructed to abstain from coffee, alcohol, nicotine, and physical exercise for at least two hours prior to the assessment. All evaluations were conducted by investigators blinded to participants’ clinical information to minimize potential measurement bias.

### 2.4. Statistical Analysis

Descriptive statistics were used to characterize the sample, with measures of central tendency and dispersion reported as appropriate. Normality was assessed using the Kolmogorov–Smirnov test. Data were stratified by sex (men and women) and expressed as mean ± standard deviation (mean ± SD) or median and interquartile range (IQR), depending on distribution. Comparisons between sexes were performed using Student’s *t*-test or the Mann–Whitney U test, as appropriate.

For comparisons of MIP and MEP within and between sex groups, participants were categorized into predefined age groups. Given the non-parametric distribution of MIP and MEP values across age strata, the Kruskal–Wallis test was applied. Additionally, to identify differences in MIP and MEP medians both within groups and between men and women, pairwise comparisons were conducted using the Mann–Whitney U test. Multiple comparisons were adjusted using the Bonferroni post hoc correction to minimize the likelihood of Type I errors.

Additionally, multivariate linear regression models were used to analyze the influence of clinical factors on MIP and MEP in the total sample. The independent variables considered for inclusion in the models were selected based on theoretical relevance and subsequently entered according to statistical criteria. Only variables that demonstrated a significant correlation with the outcome variables (*p* < 0.05), as determined by Spearman’s correlation coefficient, were retained in the regression models.

Effect sizes were calculated using Cohen’s d, a standardized measure that evaluates the magnitude of differences between groups independently of sample size. In this study, effect sizes are reported as U (effect d). The magnitude of d was classified according to established thresholds: insignificant (0.0 < d < 0.2), small (0.2 < d < 0.5), medium (0.5 < d < 0.8), and large (d > 0.8). These classifications were applied to all relevant comparisons to complement statistical significance and provide insight into the practical relevance of the observed differences.

The final linear regression models for MIP and MEP were constructed using the Stepwise method, based on the independent variables that demonstrated significant associations with each outcome. To verify the adequacy of the models and the validity of the linear assumptions, the following criteria were assessed: independence of residuals, absence of multicollinearity among independent variables, absence of influential outliers, normal distribution of residuals, homoscedasticity, and the presence of a linear relationship between dependent and independent variables. For improved model fit, potential outliers were examined and excluded when justified.

Blind data analysis was performed by an independent researcher. All statistical analyses were conducted using the Statistical Package for the Social Sciences (SPSS), version 16.0 (Chicago, IL, USA), with a significance level of 5%.

## 3. Results

**Figure 1:**
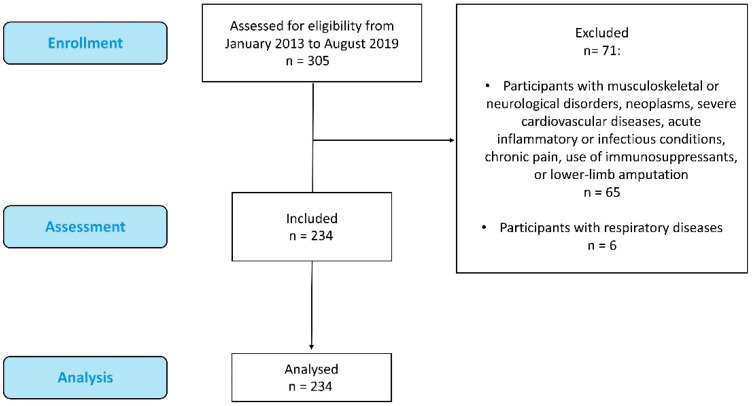
Flow chart of study participants.

In total, 305 individuals (men and women aged 30 to 80 years) were screened for eligibility. Of these, 71 were excluded for not meeting the study’s inclusion criteria—6 due to respiratory and/or pulmonary diseases and 65 for other exclusion reasons, as shown in Figure 1. The final sample consisted of 234 participants, comprising 109 women and 125 men (Figure 1).

### 3.1. Clinical and Anthropometrics Characteristics of Participants

Table 1 summarizes the anthropometric and physiological characteristics of the 234 participants, of whom 53.5% (*n* = 125) were men and 46.5% (*n* = 109) were women. The median age of the total sample was 64 years, and the median BMI was 28.66 kg/m^2^, classifying the participants as pre-obese according to World Health Organization criteria [15]. This classification was consistent across both sexes.

The mean duration of HTN diagnosis in the sample was 26 ± 8 years. The median systolic and diastolic blood pressure values were 130 mmHg (range: 90–190) and 80 mmHg (range: 50–140), respectively. Male participants exhibited slightly higher BMI values than females but presented lower blood pressure values. The mean waist circumference of the total sample was 98.67 ± 12.24 cm, with no statistically significant difference between sexes. According to the ESC/ESH Guidelines for the Management of Arterial Hypertension [18], the median blood pressure values of the sample fall within the high-normal category. Additionally, men demonstrated higher median values for both body mass and height compared with women.

Regarding respiratory muscle strength, men exhibited higher median values for both maximal inspiratory pressure (MIP) and maximal expiratory pressure (MEP) compared with women (Table 1). Median MIP values were 100 cmH_2_O (range: 20–140) in men and 80 cmH_2_O (range: 10–120) in women, whereas median MEP values were 118 cmH_2_O (range: 20–130) in men and 90 cmH_2_O (range: 20–120) in women.

### 3.2. Frequency and Percentages for Categorical Variables

In addition to hypertension, Table 2 presents the distribution of other cardiovascular and related comorbidities within the study sample. Among participants, 1.71% had a diagnosis of heart failure and 0.43% had obstructive sleep apnea. Furthermore, 5.13% reported a history of acute myocardial infarction, 2.99% had undergone coronary artery bypass grafting (CABG), and one participant (0.43%) was a permanent pacemaker user. These findings confirm that hypertension was the most prevalent cardiovascular condition in this study.

Regarding the medications used by the participants, Table 2 shows that angiotensin II receptor antagonists (ARAs II, 41%) and β-adrenoceptor antagonists (β-blockers, 28.2%) were the most frequently prescribed drug classes. Thiazide diuretics (22.2%), angiotensin-converting enzyme (ACE) inhibitors (18.8%), and calcium channel blockers (CCBs, 12%) were also commonly used.

When evaluating cardiovascular risk factors, it was observed that 84.19% of participants did not adhere to a healthy diet. Although 53.42% were former smokers, none of the participants were current smokers. Additionally, 23.93% of participants reported moderate alcohol consumption, and 8.12% identified as former alcohol users (Table 2).

Overall, the results suggest a considerable degree of homogeneity between men and women for most variables presented in Table 2, although male participants consistently exhibited slightly higher percentages, regardless of whether the response category was affirmative or negative.

### 3.3. Intra-Group Comparison Between Measured and Predicted Maximal Pressures

Table 3 presents the median MIP and MEP values observed in males and females, along with their corresponding predicted reference values. Median MIP values for both sexes were generally comparable to the predicted estimates, except for males aged 30–80 years, whose observed values were slightly lower than predicted [100 (20–140) vs. 105.7 (84.9–125.7)]. In contrast, median MEP values exceeded the predicted reference values in males aged 30–60 years [120 (20–120) vs. 119.9 (116.7–132.9)] and 30–80 years [120 (20–130) vs. 115.1 (94–135.3)], as well as in females aged 61–80 years [92.5 (20–120) vs. 81.4 (79–88.8)] and 30–80 years [90 (20–120) vs. 76 (66.2–97.3)].

### 3.4. Comparison of Maximal Inspiratory and Expiratory Pressure Values Within and Between Male and Female Groups Across Different Age Ranges

Subsequently, MIP and MEP values were compared across age groups, both within each sex and between sexes. In intragroup analyses, male participants aged 41–50 years demonstrated significantly higher median MIP values compared with those aged 61–70 years (*p* = 0.009) and those over 70 years (*p* = 0.028) (Table 4). Among females, intragroup comparisons revealed that participants aged 30–40 years had significantly higher median MIP values than all other age groups (Table 4). Between-sex comparisons showed a significant difference only among participants over 70 years of age (*p* = 0.017), with males displaying higher MIP values.

Similarly, for MEP, intragroup analyses indicated that males aged 41–60 years had significantly higher median values compared with those in the 61–70-year (*p* = 0.039) and >70-year (*p* = 0.002) age groups (Table 4). In contrast, no significant differences in median MEP values were observed across age groups among females. Between-sex comparisons demonstrated that males exhibited significantly higher median MEP values in the 51–60-year (*p* = 0.002), 61–70-year (*p* = 0.002), and >70-year (*p* = 0.001) age ranges (Table 4).

### 3.5. Factors Associated with MIP and MEP, Bivariate and Multivariate Analysis

Table 5 shows the results of the multivariate linear regression analyses for the dependent variables MIP and MEP. The final multiple regression models allowed the prediction of both pressures based on the combined influence of three explanatory variables.

In the bivariate analysis, the following predictors were significantly correlated with MIP: age (–0.262), sex (–0.251), BM (0.290), height (0.192), BMI (0.210), WC (0.242), pMIP (0.341), pMEP (0.337) and MEP (0.617). Similarly, significant correlations with MEP were observed for age (–0.248), sex (–0.415), BM (0.287), height (0.323), WC (0.172), CABG (0.100), pMIP (0.466), pMEP (0.466) and MIP (0.617). All variables showing significant correlations were initially included in their respective regression models.

During the regression modeling process, variables that did not remain significant were sequentially removed. For the MIP model, the following variables were excluded: sex, BM, height, and BMI. For the MEP model, the variables excluded were age, sex, BM, height, and WC. The final predictors retained in each model are presented in Table 5.

The final regression equation for MIP yielded F(3,201) = 68.090, *p* < 0.001, with an R^2^ of 0.495, indicating that approximately 49% of the variability in MIP was explained by the three predictors included in the model. Among these predictors, MEP (37.3%), WC (4.7%), and age (7.6%) were significantly and independently associated with MIP in the sample.

Similarly, the final regression equation for MEP resulted in F(3,201) = 77.217, *p* < 0.001, with an R^2^ of 0.528, demonstrating that approximately 53% of the variability in MEP was explained by the three predictors in this model. The variables significantly and independently associated with MEP were MIP (46.8%), pMEP (4.9%), and CABG (1.1%), as shown in Table 5.

## 4. Discussion

The present study established reference values for RMS, assessed through MIP and MEP, in individuals with hypertension, stratified by age group and sex. In addition, the findings indicate that systemic arterial hypertension does not significantly affect inspiratory or expiratory muscle strength; however, RMS appears to be influenced by both age and sex.

To the best of our knowledge, this is the first study to establish RMS reference values specifically for individuals with hypertension. These results are clinically relevant not only for the assessment of hypertensive patients but also for individuals with other conditions—such as respiratory or neuromuscular disorders—by providing reference parameters that may assist in evaluating respiratory function and guiding the prescription of appropriate RMS training intensity.

The sample in the present study consisted of individuals classified as having high-grade hypertension according to the most recent Guidelines of the European Society of Cardiology [18]. Additionally, participants were categorized as pre-obese and presented waist circumference values consistent with moderate cardiovascular risk stratification [19,20]. Given this cardiovascular risk profile, assessment strategies such as the evaluation of RMS become particularly important. Such assessment not only quantifies the degree of respiratory muscle weakness but also provides a foundation for implementing targeted interventions—most notably respiratory muscle training—which has been shown to contribute effectively to the management of hypertension.

Hypertension may compromise respiratory muscle strength through a combination of vascular, structural, metabolic, and neuromuscular mechanisms. Chronic hypertension is associated with arterial stiffening, endothelial dysfunction, and structural vascular remodeling, which can impair skeletal and respiratory muscle perfusion and oxygen delivery, ultimately affecting muscle contractile capacity and endurance [21,22]. In addition, elevated blood pressure has been linked to increased oxidative stress, chronic low-grade inflammation, disturbances in electrolyte homeostasis, and impaired muscle protein synthesis—all of which contribute to sarcopenia, muscle atrophy, and reduced muscle quality and strength, including that of the respiratory muscles [21,22,23,24,25]. Although direct investigations in hypertensive populations remain limited, evidence from individuals with vascular or cardiopulmonary conditions supports the notion that vascular pathology and systemic metabolic alterations may negatively influence RMS.

Age further exacerbates these effects. With advancing age, there is a progressive decline in muscle mass, a reduction in muscle fiber size—particularly type II fibers—along with impairments in neuromuscular activation and motor unit recruitment. These changes occur in parallel with decreased chest wall compliance, reduced lung elastic recoil, and age-related diaphragm atrophy or structural remodeling [22,23,26,27]. Collectively, these alterations contribute to reductions in both MIP and MEP [23,26,27]. When hypertension is present, age-related vascular changes—such as increased arterial stiffness, impaired baroreflex sensitivity, and endothelial dysfunction—may further compound the decline in respiratory muscle function [28].

Sex differences also play a critical role. Men generally exhibit higher RMS than women, a pattern attributed to greater overall muscle mass, larger thoracic dimensions, higher lung volumes, and more favorable chest wall mechanics [26,27,29,30]. In contrast, women—particularly those who are postmenopausal—may experience hormonal alterations that contribute to increased vascular stiffness, reduced muscle quality, and less efficient neuromuscular recruitment, which can further accentuate the negative impact of hypertension on RMS [26,27,29,30].

In addition, recent evidence suggests that hypertension may impair lung mechanics beyond its systemic vascular effects. For example, a large Brazilian study demonstrated that elevated blood pressure is associated with increased airway resistance and structural “hardening” of the bronchi, which collectively contribute to reduced respiratory capacity in older hypertensive adults [28]. Such alterations in airway and lung mechanics may further increase the workload imposed on the respiratory muscles, reduce their mechanical efficiency, and ultimately contribute to lower MIP and MEP values—particularly among older individuals with hypertension.

Although MIP and MEP values were generally higher in hypertensive men, the present study found that hypertensive women exhibited MIP and MEP values above the expected range, particularly within the 30–80-year age group. While women typically present lower absolute RMS than men, the values observed in this sample were comparable to—or even exceeded—the reference values for their sex and age. These findings suggest that hypertensive women may preserve respiratory muscle function at levels higher than anticipated. This observation is consistent with previous evidence indicating that women tend to maintain a more favorable overall health profile than men, potentially due to greater engagement in preventive healthcare measures and adherence to health-promoting behaviors [31].

When comparing the reference values identified in this study with those reported previously, our findings reveal both similarities and noteworthy discrepancies. Although the values obtained among hypertensive individuals were generally close to those predicted for healthy populations, some age groups differed from those reported by Araújo et al. [32], who established reference values for healthy Brazilian adults and observed higher MIP and MEP measurements. Conversely, another investigation involving a smaller sample of healthy Brazilian individuals reported MIP and MEP values that were similar to those observed in the present study [33].

However, it is important to emphasize that the predicted-value comparisons in the present study may not fully reflect the characteristics of the available predictive models.

In addition, these comparisons were based on a restricted population receiving care in an outpatient cardiovascular rehabilitation setting, which may limit generalizability.

A study conducted by Pessoa et al. [33] also demonstrated a strong positive correlation between self-reported levels of regular physical activity, maximal aerobic power, and RMS. This association is clinically relevant, as physical activity is known to influence both inspiratory and expiratory muscle performance. Although this variable was not directly assessed in the present study, all participants reported very low levels of habitual physical activity, which may have contributed to the RMS values observed in the present study.

In contrast, Costa et al. [34], using the same assessment apparatus and predictive equations, reported considerably lower MIP and MEP values compared with both the present study and previous investigations in healthy Brazilian populations. These discrepancies may be partially attributable to methodological differences, including the specific manovacuometer model employed. Some devices used in other studies are capable of measuring pressures beyond −120 cmH_2_O for MIP and +120 cmH_2_O for MEP, which may affect measurement accuracy and limit comparability across studies.

Although the reference values observed in hypertensive individuals in the present study were close to the predicted or previously reported values for healthy populations, it is essential that MIP and MEP assessments derived from these data be widely incorporated into RMS evaluation and used to guide the prescription of respiratory muscle training in cardiac rehabilitation programs. Galdino et al. [35] reported significant increases in both MIP and MEP among hypertensive individuals following an aerobic treadmill training protocol performed twice weekly for eight weeks. Notably, these improvements in RMS were accompanied by enhanced cardiovascular fitness—demonstrated by better performance on the six-minute walk test—and were particularly associated with reductions in both systolic and diastolic blood pressure.

Beyond reinforcing the effectiveness of aerobic exercise in managing hypertension, these findings highlight the potential value of MIP and MEP testing as tools for monitoring the outcomes of respiratory muscle training, assessing gains in respiratory strength, and investigating their direct contribution to blood pressure control.

In this context, a recent meta-analysis highlighted the importance of respiratory muscle training—particularly inspiratory muscle training—in controlling blood pressure among hypertensive individuals. The study, which included 215 participants, demonstrated that inspiratory muscle training, as assessed by MIP, was effective in reducing blood pressure levels, especially when performed at moderate intensity [36].

This study has several limitations that should be considered when interpreting the findings. First, the level of physical activity was based solely on self-reported information, which indicated generally low activity levels among participants. Although informative, this approach is subject to reporting bias; the use of validated instruments would have provided a more objective and reliable assessment of habitual physical activity. Second, due to its cross-sectional design, the study cannot establish causal relationships between hypertension and reduced respiratory muscle strength. Although associations were identified, the temporal sequence and biological mechanisms underlying these relationships remain uncertain. Future longitudinal and mechanistic studies are needed to clarify causality and the pathways involved. Another limitation is the absence of a healthy control group. Although this was not the primary objective of the study, the inclusion of a comparator group would have strengthened the interpretation of the findings by enabling direct between-group comparisons and isolating the specific impact of hypertension on RMS. Additionally, the comparison of observed MIP and MEP values with predicted reference values may not fully capture the assumptions and characteristics of the predictive equations used. Such discrepancies should be considered when extrapolating or interpreting the results.

A further methodological constraint is the small number of participants in the 30–40-year age group (n = 4), which limits the robustness of age-stratified analyses and may have introduced bias in this subgroup. Finally, because the sample consisted exclusively of Brazilian individuals, the generalizability of the findings may be limited to populations with similar geographic, ethnic, and cultural characteristics. Studies involving more diverse populations are warranted to validate and extend these reference values.

In summary, the present study established reference values for MIP and MEP in individuals with hypertension—predominantly those classified with high-normal blood pressure—across different age groups and both sexes. The results demonstrated that RMS declines progressively with advancing age and is generally lower in women. These reference values provide clinically meaningful parameters for assessing respiratory function and for understanding the relationship between RMS and blood pressure regulation. Moreover, they reinforce the clinical relevance of maximal MIP and MEP values not only for evaluating baseline respiratory status but also for monitoring the outcomes of physical and respiratory muscle training in hypertensive individuals. Taken together, these findings support the use of RMS assessment as a valuable tool for preventing disease progression and optimizing cardiovascular health, and they may assist clinicians in designing individualized rehabilitation strategies aimed at improving functional capacity and overall cardiovascular outcomes in this population.

## Figures and Tables

**Table 1:** Clinical and anthropometrics characteristics of participants.

Clinical Variable	Total Sample (*n* = 234)	Male (*n* = 125)	Female (*n* = 109)	*p* Value
Age (years)	64 (30–88)	63 (37–68)	65 (30–81)	0.510
BMI (Kg/m^2^)	28.66 (17.19–56.64)	28.23 (20.48–40.79)	29.72 (17.19–56.64)	0.018 *
WC (cm)	98.67 ± 12.24	99.74 ± 10.54	97.44 ± 13.89	0.150
SBP (mmHg)	130 (90–190)	120 (100–190)	130 (90–180)	0.019 *
DBP (mmHg)	80 (50–140)	80 (60–100)	80 (50–140)	0.001 *
HR (bpm)	71 (40–140)	80.5 (53.7–120)	72 (51–112)	0.460
BM (Kg)	76 (41.1–145)	81.5 (43–177)	71.8 (41.1–145)	0.000 *
Height (cm)	1.63 (1.40–1.87)	1.68 (1.52–1.87)	1.56 (1.40–1.77)	0.000 *
MIP (cmH_2_O) †	100 (10–140)	100 (20–140)	80 (10–120)	0.000 *
MEP (cmH_2_O) †	100 (20–130)	118 (20–130)	90 (20–120)	0.000 *

BMI, body mass index; WC, waist circumference; SBP, systolic blood pressure; DBP, diastolic blood pressure; HR, heart rate; BM, body mass; MIP, maximal inspiratory pressure; MEP, maximal expiratory pressure. Values are expressed as mean ± standard deviation for WC, and as median (minimum–maximum) for age, BMI, SBP, DBP, HR, BM, height, MIP, and MEP. * Indicates a statistically significant difference between males and females (*p* < 0.05). † Indicates *n* = 207 due to missing data from participants who were unable to complete the tests (*n* = 27).

**Table 2:** Frequency and Percentages for Categorical Variables.

Categorical Variable	Male	Female	Total Sample
	*n*/%	*n*/%	*n*/%
HTN	125 (53.42)	109 (46.58)	234 (100)
HF	2 (0.86)	2 (0.86)	4 (1.71)
AMI	9 (3.85)	3 (1.28)	12 (5.13)
CABG	5 (2.14)	2 (0.85)	7 (2.99)
PPM	1 (0.43)	0 (0)	1 (0.43)
OSA	0 (0)	1 (0.43)	1 (0.43)
Diet	15 (6.41)	22 (9.40)	37 (15.81)
Smoking	Ex-S 74 (31.62)	51 (21.80)	125 (53.42)
Alcoholism	Ex-A 16 (6.84)	3 (1.28)	19 (8.12)
	40 (17.09)	16 (6.84)	56 (23.93)
**Anti-hypertensive** **drugs**			
ARA II	96 (41)	42 (18)	54 (23)
β-blockers	66 (28.2)	29 (12.3)	37 (15.8)
Thiazide diuretics	52 (22.2)	16 (6.8)	36 (15.4)
ACE inhibitors	44 (18.8)	22 (9.4)	22 (9.4)
CCBs	28 (12)	24 (10.2)	4 (1.7)
Unmedicated	35 (15)	18 (7.6)	17 (7.3)

HTN, hypertension; HF, heart failure; AMI, acute myocardial infarction; CABG, coronary artery bypass grafting; PPM, permanent pacemaker; OSA, obstructive sleep apnea, Ex-S, ex-smoker; Ex-A, ex-alcoholic; ARA, II angiotensin II receptor antagonists; β-blockers, β-adrenoceptor antagonists; ACE, inhibitors angiotensin converting enzyme inhibitors; CCBs, calcium channel blockers.

**Table 3:** Intra-group Comparison Between Measured and Predicted Maximal Pressures.

Age Group (Years)	Male		*p* Value	Female		*p* Value
	MIP	PMIP		MIP	PMIP	
30–60	118 (40–120) (*n* = 44)	110.5 (107.3–123.3) (*n* = 44)	0.160	87.5 (20–120) (*n* = 28)	83 (81–88.84) (*n* = 28)	0.92
61–80	100 (20–126) (*n* = 60)	99.3 (92.9–106.5) (*n* = 60)	0.220	80 (10–120) (*n* = 64)	76.6 (71.2–80.5) (*n* = 64)	1.00
30–80	100 (20–140) (*n* = 104)	105.7 (84.9–125.7) (*n* = 104)	0.033 *	80 (10–120) (*n* = 92)	78.6 (70.7–95.7) (*n* = 92)	0.56
	MEP	PMEP		MEP	PMEP	
30–60	120 (20–120) (*n* = 44)	119.9 (116.7–132.9) (*n* = 44)	0.000 *	92.5 (20–120) (*n* = 28)	81.4 (79–88.8) (*n* = 28)	0.160
61–80	110 (25–130) (*n* = 60)	108.6 (102.1–115.9) (*n* = 60)	0.07	84.5 (20–120) (*n* = 64)	73.5 (66.8–78.4) (*n* = 64)	0.010 *
30–80	120 (20–130) (*n* = 104)	115.1 (94–135.3) (*n* = 104)	0.000 *	90 (20–120) (*n* = 92)	76 (66.2–97.3) (*n* = 92)	0.001 *

MIP, maximal inspiratory pressure; PMIP, predicted maximal inspiratory pressure; MEP, maximal expiratory pressure; PMEP, predicted maximal expiratory pressure. * Indicates a statistically significant difference (*p* < 0.05) compared to the predicted value for the respective sex, according to the Wilcoxon test.

**Table 4:** Comparison of maximal inspiratory and expiratory pressure values intra- and between male and female groups across different age ranges.

Age Group (Years)	Male		Female		Male/Female	
	MIP Med. (Q1–Q3)	*p* Value	MIP Med. (Q1–Q3)	*p* Value	Between-Groups U (effect d)	*p* Value
30–40 ^a^	100 (80–120) (*n* = 4)	Ns	120 (110–120) (*n* = 4)	*p* = 0.007 *^,c^ *p* = 0.037 *^,d^ *p* = 0.003 *^,e^	5.0 (0.268) ^S^	*p* = 0.486
41–50 ^b^	120 (118–120) (*n* = 15)	*p* < 0.009 *^,d^ *p* < 0.028 *^,e^	120 (100–120) (*n* = 3)	ns	21.0 (0.033) ^I^	*p* = 0.912
51–60 ^c^	105 (90–120) (n = 28)	Ns	85 (60–120) (n = 25)	*p* = 0.007 *^,a^	250.0 (0.249) ^S^	*p* = 0.076
61–70 ^d^	100 (70–120) (*n* = 35)	*p* < 0.009 *^,b^	80 (57.5–105) (*n* = 35)	*p* = 0.037 *^,a^	510.5 (0.165) ^I^	*p* = 0.170
>70 ^e^	90 (70–120) (*n* = 28)	*p* < 0.028 *^,b^	70 (50–90) (*n* = 25)	*p* = 0.003 *^,a^	217.0 (0.327) ^S^	*p* = 0.017 *
**Age group** **(Years)**	**MEP** **Med. (Q1–Q3)**	***p*** **value**	**MEP** **Med. (Q1–Q3)**	***p*** **value**	**Between-Groups** **U (effect d)**	***p*** **Value**
30–40 ^a^	100 (80–120) (*n* = 4)	Ns	102.5 (85–120) (*n* = 4)	ns	6.0 (0.165) ^I^	*p* = 0.686
41–50 ^b^	120 (120–120) (*n* = 15)	*p* < 0.039 *^,d^ *p* < 0.002 *^,e^	100 (90–110) (*n* = 3)	ns	11.0 (0.422) ^S^	*p* = 0.203
51–60 ^c^	120 (97.5–120) (*n* = 28)	Ns	90 (70–100) (*n* = 25)	Ns	176.0 (0.436) ^S^	*p* = 0.002 *
61–70 ^d^	110 (90–120) (*n* = 35)	*p* < 0.039 *^,b^	90 (70–100) (*n* = 35)	Ns	365.5 (0.366) ^S^	*p* = 0.002 *
>70 ^e^	100 (85–120) (*n* = 28)	*p* < 0.002 *^,b^	80 (60–100) (*n* = 25)	Ns	172.0 (0.327) ^S^	*p* = 0.001 *

*MIP, maximal inspiratory pressure; MEP, maximal expiratory pressure;* ^a^, 30–40; ^b^, 41–50; ^c^, 51–60; ^d^, 61–70; ^e^, >70; ns, non-significant difference relative to other age groups. * Indicates a statistically significant difference (*p* < 0.05) in MIP or MEP in the within-group (men or women, Wilcoxon test) or in the between-groups (men and women) comparisons across different age ranges. “Med. (Q1–Q3)” represents the median value of the MIP or MEP variables, followed by the 1st and 3rd quartiles. The difference between them reflects the interquartile range (IQR). U (effect d), effect size classified according to significance level: I—insignificant (0.0 < d < 0.2); S—small (0.2 < d < 0.5); M—medium (0.5 < d < 0.8); L—large (d > 0.8).

**Table 5:** Factors associated with MIP and MEP, bivariate and multivariate analysis.

	MIP				MEP			
Variable	Multivariate Linear Regression				Multivariate Linear Regression			
	β	t	*p*	R^2^	β	t	*p*	R^2^
Age	−0.141	−2.760	<0.05 ^†^	0.075	__	__	__	__
WC	0.101	1.984	<0.05 ^†^	0.047	__	__	__	__
CABG	__	__	__	__	0.113	2.356	<0.05 ^†^	0.011
MIP	__	__	__	__	0.599	11.693	<0.05 ^†^	0.468
MEP	0.637	12.257	<0.01 ^†^	0.373	__	__	__	__
pMEP	__	__	__	__	0.247	4.827	<0.05 ^†^	0.049

R^2^ = adjusted coefficient of determination; β = standardized coefficients; t = t statistic; *p* = *p*-value; WC = waist circumference; CABG = coronary artery bypass graft; MIP = maximal inspiratory pressure; MEP = maximal expiratory pressure; pMEP = predicted maximal expiratory pressure. ^†^ = significant independent association in the regression model adjusted.

## Data Availability

The original contributions presented in this study are included in the article. Further inquiries can be directed to the corresponding author.
